# Discrimination of familiar human faces in dogs (*Canis familiaris*)

**DOI:** 10.1016/j.lmot.2013.04.005

**Published:** 2013-11

**Authors:** Ludwig Huber, Anaïs Racca, Billy Scaf, Zsófia Virányi, Friederike Range

**Affiliations:** a Messerli Research Institute, University of Veterinary Medicine Vienna, Medical University Vienna, University of Vienna, Austria; b Clever Dog Lab, Vienna, Austria; c School of Life Sciences, University of Lincoln, UK; d School of Psychology, University of Lincoln, UK

**Keywords:** Face discrimination, Face recognition, Internal features, Forced two-choice, Dogs

## Abstract

Faces are an important visual category for many taxa, and the human face is no exception to this. Because faces differ in subtle ways and possess many idiosyncratic features, they provide a rich source of perceptual cues. A fair amount of those cues are learned through social interactions and are used for future identification of individual humans. These effects of individual experience can be studied particularly well in hetero-specific face perception. Domestic dogs represent a perfect model in this respect, due to their proved ability to extract important information from the human face in socio-communicative interactions. There is also suggestive evidence that dogs can identify their owner or other familiar human individuals by using visual information from the face. However, most studies have used only dogs’ looking behavior to examine their visual processing of human faces and it has been demonstrated only that dogs can differentiate between familiar and unknown human faces. Here, we examined the dog's ability to discriminate the faces of two familiar persons by active choice (approach and touch). Furthermore, in successive stages of the experiment we investigated how well dogs discriminate humans in different representations by systematically reducing the informational richness and the quality of the stimuli. We found a huge inter-individual and inter-stage variance in performance, indicating differences across dogs in their learning ability as well as their selection of discriminative cues. On a group level, the performance of dogs significantly decreased when they were presented with pictures of human heads after having learned to discriminate the real heads, and when – after relearning – confronted with the same pictures showing only the inner parts of the heads. However, as two dogs quickly mastered all stages, we conclude that dogs are *in principle* able to discriminate people on the basis of *visual* information from their *faces* and by making active choices.

In the last decade a plentitude of studies has been devoted to the investigation of the socio-cognitive skills of dogs and the reasons for their special capacity to communicate and form relationships with humans (reviewed in Coppinger and Coppinger, 2001, Miklósi, 2007). These studies have provided evidence for high levels of attentiveness toward human behavior, enhanced both by phylogenetic and ontogenetic processes (Gácsi et al., 2009a, Hare and Tomasello, 2005, Marshall-Pescini et al., 2009, Virányi et al., 2008), and a very flexible sensitivity for salient human communicative cues (Gaunet, 2008, Elgier et al., 2009, Horn et al., 2012). As one conclusion from those studies, it has been assumed that the anthropogenic selective environment has affected behavior systems in dogs that support the recognition of humans as social partners (Gácsi et al., 2009a, Hare et al., 2002). Positive feedback between evolutionary (selective) and ontogenetic processes are thought to have contributed to the increased readiness of dogs to look at the human face, providing the basis for complex forms of dog-human communication (Gácsi et al., 2009b, Miklósi et al., 2003). By monitoring human faces, dogs seem to obtain a continuous stream of social information, ranging from communicative gestures to emotional and attentive states (Call et al., 2003, Gácsi et al., 2004, Miklósi et al., 1998, Schwab and Huber, 2006, Soproni et al., 2001).

Other recent studies provided both indirect and direct evidence that dogs extract a sufficient number of cues from the head or the face of humans to be able to differentiate between them or even to recognize familiar persons. Indirect evidence comes from an attention study in which the lack of visual access to the person's head affected the behavior of the dogs (Mongillo, Bono, Regolin, & Marinelli, 2010). In particular, the dogs’ attention toward their owner was significantly lower when the latter was wearing a hood covering her/his head. Direct evidence comes from four looking preference studies. Firstly, dogs looked longer at pictures of upright novel (vs. familiar) human faces, indicating that they can differentiate individual humans on the basis of visual facial cues alone (Racca, Amadei, Ligout, Guo, Meints, & Mills, 2010). Secondly, dogs showed a left gaze bias toward both negative and neutral expressions, but not toward positive expressions of human faces (Racca, Guo, Meints, & Mills, 2012). Thirdly, dogs looked longer at the face of their owner when presented just after the voice of another person (a stranger) rather than the voice of the owner (calling them) (Adachi, Kuwahata, & Fujita, 2007). This suggests that dogs actively generate their internal representation of the owner's face when they hear him/her calling them. Finally, domestic dogs demonstrated a human-like *left gaze bias*, accounting for a right hemisphere dominance, toward human faces but not toward monkey or dog faces (Guo, Meints, Hall, Hall, & Mills, 2009). Altogether, these studies suggest that the features of the human head or face represent a primary element during the visual search for familiar humans in dogs.

Dogs may not be special in using faces for recognition and communication purposes. Faces are an important category of visual stimuli for animals in all major vertebrate taxa, possibly reflecting the early emergence of neural specialization (expert specialist mechanisms) for faces in vertebrate evolution (Leopold & Rhodes, 2010). For instance, primates may have evolved special abilities for reading faces due to their complex social life (e.g. Marechal et al., 2010, Parr and de Waal, 1999, Parr et al., 2000). In contrast, faces may merely be a category of objects that have a common configuration, and subtle variations in them are identified through learning and individual experiences (Diamond & Carey, 1986). In line with this is the ability of non-social species, such as crayfish, to identify the faces of fight opponents (Van der Velden, Zheng, Patullo, & Macmillan, 2008) and the ability of sheep and cattle, social species, to visually recognize faces of conspecifics, although their social life may not be as complex as that of primates (Coulon et al., 2007, Coulon et al., 2009, Kendrick et al., 1995, Kendrick et al., 2001). Especially sheep have shown astonishing competences of face perception and discrimination. They also distinguish visually between different breeds of sheep, between genders within their own breed and even between individual ewes (Kendrick et al., 1996, Peirce et al., 2000). They can recognize individual conspecifics on a computer screen, and they can do so even when images are presented at a small scale or when identity information is reduced (Tate, Fischer, Leigh, & Kendrick, 2006).

While conspecific face recognition seems to be widespread in the vertebrate kingdom, evidence for recognition of heterospecifics is scarce. Heterospecific recognition is supposed to be beneficial especially during predation, which includes the recognition of humans by wild animals in urban environments (e.g. Bogale et al., 2011, Ferrari et al., 2008, Lee et al., 2011, Levey et al., 2009, Marzluff et al., 2010, Slobodchikoff et al., 1991, Stone, 2010). A special case, of course, is the recognition of humans in farm livestock or pets with close bonding to human caretakers (Racca et al., 2010, Stephan et al., 2012, Taylor and Davis, 1998). For instance, sheep are capable of discriminating between various photographically represented faces of dogs, humans and goats (Kendrick et al., 1995) and can even recognize the faces of individual human caretakers and sheep dogs (Davis et al., 1998, Da Costa et al., 2004), although they are more competent with pictures of conspecifics than heterospecifics (Peirce, Leigh, daCosta, & Kendrick, 2001).

Undoubtedly, such abilities are possible only with a fair amount of experience with the other species. According to the “pre-exposure” hypothesis (Lee et al., 2011), all urban living species with much exposure to humans should rapidly learn to discriminate among humans. In non-human primates raised in close contact with humans, this effect may be so strong that it converts their face recognition abilities. Chimpanzees raised in a human environment showed a superior ability to discriminate among pictures of unknown humans over unknown chimp faces (Martin-Malivel & Okada, 2007). Similar effects of individual expertise have also been reported from rhesus macaques (Leopold, Bondar & Giese, 2005) and Japanese macaques (Sugita, 2008). However, the faces of primates may be similar enough to generate such cross-species effects. This is not the case with sheep. Their faces are very different from human faces, but they could still identify human faces and showed a small inversion-induced decline in discriminatory performance (Peirce et al., 2001). Importantly, their ability to distinguish human faces was shown with individuals that had a great deal of close visual contact with humans (on average 2–3 h per day for 3 years). Still, this is not comparable to pet dogs, which may hold the most intense relationship with humans among non-human animals. Pet dogs thus have lots of experience with human faces, probably more than with conspecific faces.

It is important to note that, instead of being able to *recognize* a person, perceivers may only be able to regard them as *familiar*. None of the former studies examining face discrimination in dogs can differentiate between these two explanations because they asked dogs to discriminate between a familiar and an unknown face (Adachi et al., 2007, Racca et al., 2010). Individual recognition refers to the ability to identify an individual by using its individually distinctive characteristics, i.e. by unique recognition cues that were learned during past interactions (Tibbets & Dale, 2007). It is likely that this ability is based on the default mechanisms of discrimination learning, i.e. learning to attend to those perceptual features that distinguish the target from the distractors (or the S+ from the S−). These diagnostic features enable identification but can also be used for categorization and concept formation (for a review, see Huber, 2000, Huber, 2010). Feature learning is the key for flexible switching between different perceptual problems, as has been convincingly shown in pigeons (Huber & Aust, 2011).

In many experiments on discrimination or recognition of individuals or faces, photographic stimuli have been used, usually presented on computer screens. This generates a further complication. Conclusions in terms of individual recognition or only familiarity would require us to be able to know whether the animals recognize that the photographs ‘represent’ real-life individuals. In fact, to see that a picture represents a real-life object is not a simple task. It requires dual representation; that is, an organism must mentally represent both the symbol itself and its relation to the referent (DeLoache, 2000). This form of representational insight has been shown in only a small number of mammals (e.g. Aust and Huber, 2006, Boysen and Berntson, 1989, Dasser, 1987, Kendrick et al., 1996, Parr and de Waal, 1999, Pokorny and de Waal, 2009).

The present set of experiments investigated the ability of domestic dogs to discriminate between two familiar humans. One purpose of this work was to examine basic perceptual questions. Can dogs discriminate (familiar) humans on the basis of the visual features of their faces alone (Stages 2 and 3) or do they require more visual (rest of the body) or other sensory information, like olfaction (pre-training and Stage 1)? If they can do so with visual information alone, as has been indicated in previous experiments, what visual features would dogs use to accomplish this? Can they make the discrimination on the basis of the faces only (Stage 3) or do they need other parts of the head (Stage 2)? As we required them to discriminate between familiar people, the discrimination cannot be based on familiarity vs. novelty. It could be facilitated by individual recognition, however. Nevertheless, testing for individual recognition was not the aim of this study because the same two faces were used throughout the experiment, and successful discrimination was possible also by relying on one or a few visual cues.

A second purpose of this study was to examine procedural questions, i.e. the ability of dogs to discriminate the faces of (different) humans in a two-choice paradigm. So far, by means of dogs actively making a choice, discrimination learning has been shown with images of dogs and landscapes presented on a computer screen (Range, Aust, Steurer, & Huber, 2008) and with images of the *same* person in two different emotional states (smiling versus neutral) (Nagasawa, Murai, Mogi, & Kikusui, 2011). The discrimination between *different* human persons has been indicated only in a passive manner by looking preference studies (Adachi et al., 2007, Racca et al., 2010). Therefore, the task for the dogs in the present study was to make the decision explicit by approaching the positively assigned human (S+) and touching its face. We examined dogs’ ability to solve this discrimination task at three levels of increasing difficulty.

## Methods

### Ethics statement

All procedures were performed in compliance with relevant laws and institutional guidelines. The owners participated in this study on a voluntary base. The owners signed a consent form and agreed to have their portraits published in this paper. The daily testing procedure was short and entirely non-invasive. No special permission for use of animals (dogs) in such socio-cognitive studies is required in Austria.

### Subjects

Dogs (*N* = 15) and their owners were recruited to participate in this study at the Clever Dog Lab in Vienna, Austria, between February and December 2011. Only dogs older than 2 years were tested and various breeds were included (see Table 1). Prior to the study all dogs had lived as pets with their owners since they were between nine weeks and one year old. Dogs were pseudo-randomly assigned to either the *Owner+* group (*N* = 8) or the *Owner*− group (*N* = 7). The two groups were balanced for sex, age, breed and experience with clicker training as much as possible (Table 1).

**Table 1:** Specifications of dogs participating in the study.

Group	Name	Breed	Age (years)	Sex	Owner	Non-Owner	Clicker trained	Familiarity non-owner (years)	Meeting frequency non-owner (per week)
Owner+	Aico	Doberman	3	M	Manon	Xenia	No	2	2
	Caya	Border Collie	4	F	Christina	Christa	No	4	1
	Flamme	Berger des pyrenees	3	M	Ulli	Birgit	Yes	2	2
	Ivi	Border Collie	10	F	Christa	Christina	No	3.5	1
	Jock	Border Collie	4	M	Christa	Christina	No	3.5	1
	Loki	Mix	5	F	Xenia	Manon	Yes	2	2
	Lucy	Rottweiler	6	F	Birgit	Ulli	Yes	2	2
	Marty	Mix	7	M	Marion	Julia	Yes	3	2.5

Owner−	Baris	Border Collie	6	M	Christina	Christa	No	4	1
	Cap	Border Collie	4	M	Christa	Christina	No	3.5	1
	Flag	Australian sheperd	2	M	Xenia	Manon	Yes	1	2
	Ivy	Doberman	2	F	Manon	Xenia	No	1.5	2
	Jamil	Mix	7	M	Julia	Marion	Yes	3	2.5
	Leah	Border Collie	7	F	Christa	Christina	No	3.5	1
	Nessie	Mix	10	F	Ulli	Birgit	Yes	2	2

### Experimental setup

**Fig. 1:**
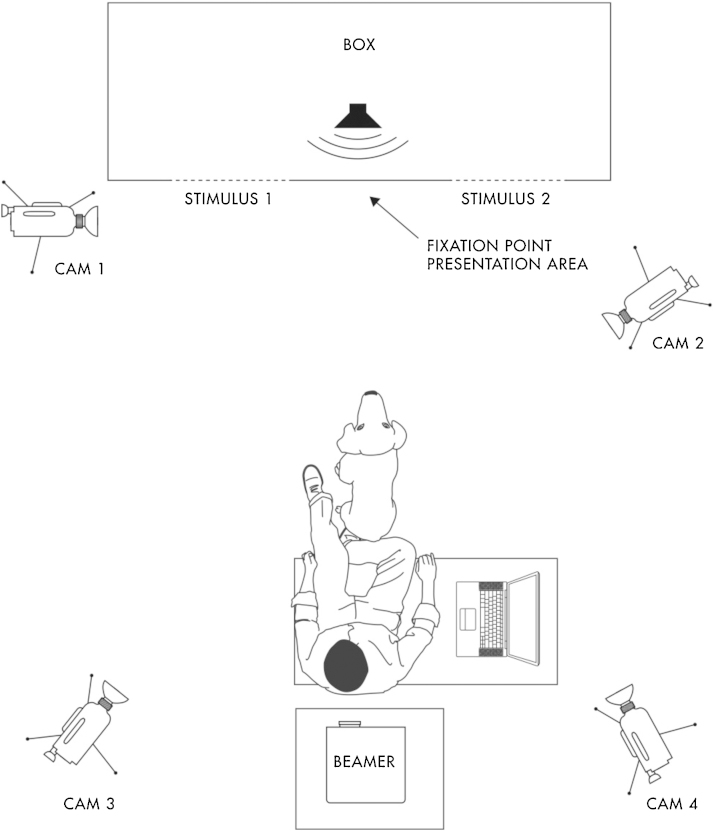
Sketch of the experimental setup (for details see text), seen from above.

All tests were conducted in the experimental room (5 m × 6 m) of the ‘Clever Dog Lab’ in Nussgasse 4, 1090 Vienna. A box large enough to hold two adult, kneeling people (150 cm × 75 cm × 105 cm) was positioned in front of the wall (Fig. 1). Its front side was covered with a white sheet that had two identical holes (15 cm × 20 cm), 75 cm apart and at a height of 65 cm. In the case of small dogs, two small boxes (50 cm × 25 cm × 25 cm) were added in front of the two holes so that the dogs could reach the heads of the people kneeling inside.

The experimenter sat on a chair with the dog between his legs at a distance of 1.5 m from the center of the box, facing it. In the case of two dogs, the experimenter sat on the floor behind the dog to make those dogs more comfortable. The pictures were presented by a data projector (beamer) which was positioned behind the experimenter at a height of 1.5 m. Next to the experimenter was a chair with a laptop computer. The computer was linked to a speaker positioned inside the box (center) and to the projector and used to control the stimuli.

We used four cameras to record the behavioral response of the dog. The first camera was facing toward the front, left side of the box (Cam1), recording whether the dog touched the face of the person or not. The second camera (Cam2) was set-up to the right side of the box and recorded the experimenter, the dog and the area in front of the box. Two cameras were positioned on the two sides of the projector, recording the events in the entire room (Cam3) and only at the box (Cam4). For effective projection of pictures, the lights were switched off and two of three windows were covered with a curtain. A PC in the neighboring room was used for the video recording.

## Procedure

We applied a two-way conditioned discrimination procedure, in which one of the two stimuli was consistently associated with a food reward. According to the group assignment, the subjects were trained to either touch the face of their owner (*Owner+ group)* or the face of another person, also familiar to the dog (*Owner− group*). The latter person was a close friend of the owner who regularly met the dog (see Table 1 for information about the frequency of meetings). Both persons were of the same sex and of similar age and were not allowed to wear heavy make-up, face piercings or glasses during the experiments. Due to a great majority of female owners in our database, only women were involved in the study.

The experiment consisted of pre-training followed by three stages of discrimination training that differed only in the stimuli presented. We reduced in a stepwise manner both the quality and the quantity of information available to make the discrimination.

The pre-training as well as the three different discrimination stages consisted of several sessions of 10 trials each. A maximum of three sessions per dog were conducted per day with a 5-min break between sessions. The side of the presentation of the owner was semi-randomized so that no more than two trials were conducted in a row with the owner being on the same side. The criterion for completing a stage and passing to the next one was set at 70% correct choices in three consecutive sessions (corresponding to *p* ≤ 0.043, binomial test). Nevertheless, we made sure that the third successful session was conducted on a different day than the previous two so that the last successful session could be immediately followed by the first session of the following stage. This was done in order to evaluate generalization from one stage to the next, controlling for daily differences in attention or motivation.

### Pre-training

The aim of the pre-training was to familiarize the dog with the head/face discrimination tasks. The subjects were progressively trained to touch either the owner's face or the other familiar person's face (depending on their group assignment), while both were sitting 50 cm apart in front of the box. The dogs were trained by encouraging them using a happy voice and by giving a treat (a small commercial dog food pellet or, in case of some not highly motivated dogs, small pieces of sausage) after each correct choice and silence after an incorrect choice. For those subjects that were familiar with clicker training (see Table 1), we also used the clicker for the approach training (*N* = 7).

To familiarize the dogs with the task and to get them used to being controlled by the experimenter, the owner and familiar person were allowed to interact with the dog, to look at the dog and to call the dog to them. Once the dog started touching the face of the assigned person, the command “kiss”, “touch” or “face” was introduced dependent on the owner's preference. As the dogs progressively learned what they were supposed to do, the owner and familiar person stopped looking at the dog, stared straight at the opposite wall instead, stopped interacting with the dog and displayed neutral facial expressions. They also started to swap places when instructed by the experimenter. Furthermore, the dogs were trained to sit in front of the experimenter, to face the stimuli, and were released with the command introduced before. Finally, the lights were switched off and the projector was turned on projecting a white slide to which the dogs became habituated.

Once the dogs were familiar with all requirements of the discrimination task, they received standardized training sessions. Before starting the first stage of the experiment, they had to reach our success criteria for having learned the task, paid sufficient attention and performed in a stable manner. Dogs needed three to eight of these sessions to reach our success criteria. These standardized sessions started with the projection of a white slide onto the box. The experimenter, owner and familiar person entered the room first without the dog, and the experimenter directed the two people to their positions for the first trial. Once the owner and familiar person took positions sitting crossed legged on the floor in front of the box, the experimenter fetched the dog, holding it on the collar, and asked it to sit down at the starting point facing the stimuli. From that moment on, the owner and familiar person were asked to look straight at the opposite wall, to have neutral facial expressions and to avoid any interaction with the dog.

When the dog was at the starting point facing the box, the experimenter presented a sound from a speaker positioned in the middle of the box to attract the dog's attention and simultaneously presented a fixation point in the middle of the box between the two holes in order to centralize the dog's gaze. For the sound, we used standard non-animal sounds from the PowerPoint program 2007 (e.g. laser guns, flipping coins, passing train). The sound was varied between trials to avoid dogs habituating toward the sound. As a fixation point, we used a red dot that started small and grew to twice its size repeatedly. The fixation point was visible as long as the sound was presented. Although the experimenter was looking toward the floor, he/she could see the dog's head direction peripherally (but not the people's faces nor the pictures projected on the screen). If the dog was looking straight ahead, it was assumed that it was centralized, and the experimenter switched the projection to the stimuli and released the dog after 3 s with the trained command. In some cases, the experimenter had to repeat the command, slightly push the dog when it remained sitting or standing after the command was given or point toward the middle of the box so that the dog would make a choice. During the entire time of handling the dog, the experimenter looked straight at the ground to minimize involuntary cueing. However, in the training stage and to a lesser degree in stage 1 (see below), the experimenter might have been aware of the position of the people, which might have led to involuntary cueing (but see Schmidjell, Range, Huber, & Virányi, 2012 for the difficulty of actually cueing dogs in such experiments). If the dog made the correct choice, the experimenter acknowledged the choice by clicking the clicker or praising the dog (“super!”), called the dog back and rewarded it with a food reward. If the dog made the wrong choice, the experimenter called the dog back straight away and did not reward the dog. After the dog was positioned again in the starting position, the next trial would start.

The experimenter instructed the owner and the familiar person whether to change position or not (‘stay’ or ‘change’). However, even if they stayed in the same position, they stood up and sat down again briefly so that there was always some movement as well as noise between trials.

### Stage 1: discrimination between heads in live presentation

The procedure of the first stage was identical to the pre-training with the difference that the owner and the familiar person were sitting/kneeling *inside* the box. When the experimenter stopped the sound and the projection of the dot, they simultaneously pushed their heads through holes in the front wall of the box (Fig. 1). As in the pre-training, they looked straight at the opposite wall, had neutral facial expressions and did not interact with the dog. If the dog made a wrong choice, both persons would withdraw their heads inside the box in order to prevent the dog from touching the correct (S+) face. Between each trial the owner and the other familiar person moved within the box–invisible to the dog and experimenter, whether they changed locations or not. However, even if they stayed in the same position, they briefly stood up and sat down again so that there was always some movement as well as noise between trials.

### Stage 2: discrimination between pictures of heads

**Fig. 2:**
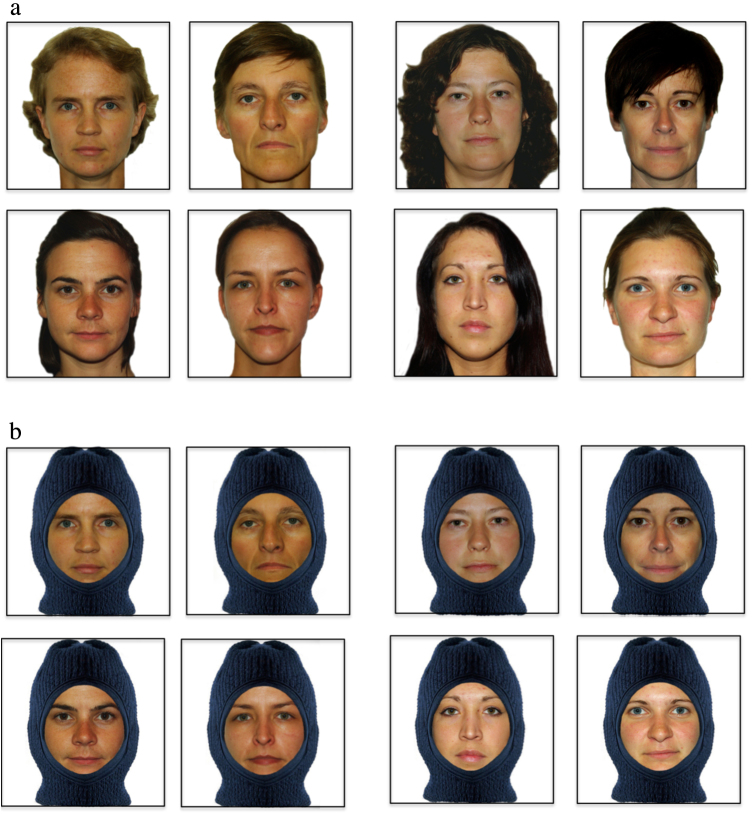
All stimulus pairs used in the Stage 2 (2a) and Stage 3 (2b) of the study (see text).

Stage 2 was identical to the previous one with the exception that instead of the heads of the real people being shown, frontal-view photographs of their heads were projected onto the box (Fig. 2a). The pictures of each pair of people had been taken in the Clever Dog Lab using a PENTAX K10 camera, from the same location and at the same time in order to get similar lightning. People were asked to look straight into the camera with a neutral facial expression (no smile) and were previously asked to wear no makeup. The pictures were then processed under Photoshop CS2 to visually adjust the lightning and contrast of the pictures and to add a homogenous white background for each of them. During the experiment, the pictures were projected onto the box at the place of the holes (on a sheet of white paper) using PowerPoint software. The sizes of the pictures were adjusted to match those of the real heads, being about a 7.6**°** viewing angle for the dogs from a 1.5 m distance.

The experimenter was blind regarding the side of the stimulus presentation due to the fact that all presentation orders were prepared several days ahead of the testing days simultaneously for several dogs and it was impossible to remember each presentation. The experimenter released the dog and then looked at the screen to see which side the S+ was presented on and to reward or not reward the dog depending on its choice. If the dog made the incorrect choice, the experimenter called the dog straight back and switched the PowerPoint presentation to a white slide in order to prevent the dog from touching the second picture.

### Stage 3: discrimination between pictures of faces (the ‘balaclava mode’)

The aim of this third, final stage was to test the dog's ability to discriminate between pictures of the internal features of the head of its owner and of the familiar person, i.e. using their face only. We used the same pictures as in Stage 2 but digitally overlaid a balaclava (ski mask) to hide the hair and the head contour, so that only the face (eyebrows, eyes, nose, cheeks and mouth) was visible (Fig. 2b). This was done instead of removing the external features digitally, which would result in a very unnatural representation of the head. The projection of the pictures was identical to Stage 2.

### Data and statistical analyses

All videos were coded using the program *Solomon Coder beta* (^©^2006–2011 András Péter). A trial started when the dog was released by the experimenter and ended with the first choice of the dog. For each trial, we coded whether or not the choice was correct. Furthermore, we coded whether or not the experimenter had to repeat the command, and had to slightly push the dog when it remained sitting or standing after the command was given. Occasionally, it happened that a dog refused to make a choice even after such encouragement. In this case, the experimenter led the dog to the correct stimulus in order to overcome such motivational problems. If leading the dog to the correct stimulus for a maximum of five trials did not improve its readiness to make a choice independently, the session was terminated and the dog was tested on another day. All of the trials with such strong helping were excluded from the analyses.

We calculated a generalized linear mixed effect model (GLMM) using the binomial distribution to investigate whether the performance of the dogs was influenced by the test stages and their group assignment. In addition, we calculated another GLMM using the binomial distribution to analyze whether there was a learning effect over the first three sessions of each stage (this number was chosen because all dogs that entered a stage had at least three sessions but not necessarily more). Furthermore, we analyzed whether reaching criterion or not was influenced by group assignment (Owner+ vs. Owner−) or stage, by calculating a GLMM using the binomial distribution. In addition, we calculated a GLMM using the Poisson distribution to investigate whether the number of sessions to reach the criterion was influenced by the factors Stage and Group. The individuals were involved as a random effect in all models.

One-sample *t*-tests were calculated to investigate whether the dogs’ performance in the first ten trials of each stage was above chance and to check for a side bias over all trials. However, to look for an individual side bias, we calculated binomial tests. The analyses were done with the statistical package R 2.15.0.

## Results

### Overall performance (reaching criterion)

All 15 dogs that participated in the study mastered the pre-training, with an average of 92.67% correct choices in the last pre-training session. Only one dog failed to reach criterion in Stage 1 (Ivy), in which only the heads of the humans were visible. However, in this stage only four of the successful dogs solved the task in the minimum number of sessions; the others needed 5–17 sessions. In Stage 2, four further dogs failed to pass the criterion in a reasonable number of sessions or were discontinued because of motivation problems. Finally, from the 10 successful dogs of Stage 2, only two (Caya and Baris) mastered the last stage when only the internal features of the faces were available (see Table 1). Whether a dog reached the criterion or not was not influenced by its group assignment (Owner+ vs. Owner−) (GLMM: *z* = −0.01, *p* = 0.99). Statistically, there was no difference between Stages 1 and 2 in the number of dogs reaching criterion (GLMM: *z* = 1.49, *p* = 0.14, after Holm–Bonferroni correction *p* > 0.05), but dogs were more likely to reach the criterion in both Stage 1 (GLMM: *z* = −3.369, *p* = 0.001, after Holm–Bonferroni correction *p *≤ 0.05) and Stage 2 than in Stage 3 (GLMM: *z* = −2.787, *p* = 0.005, after Holm–Bonferroni correction, *p *≤ 0.05).

While the dogs (with exception of Ivy) needed on average only few sessions to complete Stage 1 (6.5 sessions), the successful dogs needed a mean of 14.4 sessions to reach criterion in Stage 2. The two dogs that discriminated correctly between the internal features of the faces in Stage 3 performed surprisingly well in the very first three sessions. Both made 70% or more correct choices in the first two sessions and made 21 correct choices in the first three sessions, corresponding to performance significantly above chance (*p *≤ 0.043, binomial test). Caya did not immediately reach criterion, however, because she had only six correct choices in the third session and then needed ten more sessions to reach criterion. We also compared statistically the number of sessions the dogs needed to reach criterion across stages. Since only two dogs reached the criterion in the last experimental stage, we compared only Stages 1 and 2. Overall, while we found no influence of the group assignment on the number of sessions needed to reach the criterion (GLMM: *F*_13_ = 0.02, *p* = 0.90), the dogs needed more sessions to reach the criterion in Stage 2 than in Stage 1 (GLMM: *z* = −5.432, *p* < 0.001) (Table 2).

**Table 2:** Number of sessions required to reach criterion (see text) or until the termination (in parentheses).

Group	Name	Experimental stages		
		Stage 1	Stage 2	Stage 3
Owner+	Aico	6	(21)	–
	Caya	3	8	13
	Flamme	7	20	(30)
	Ivi	3	6	(25)
	Jock	5	–	–
	Loki	3	9	(29)
	Lucy	3	14	(25)
	Marty	8	(35)	–

Owner−	Baris	8	4	3
	Cap	5	6	(20)
	Flag	6	14	(25)
	Ivy	(20)	–	–
	Jamil	11	36	(26)
	Leah	6	–	–
	Nessie	17	27	(25)

### Number of correct choices across the 3 stages

We found an overall difference between the three stages based on the number of correct choices out of all trials. The dogs made more correct choices in Stage 1 than in Stage 2 (GLMM: *z* = 5.646, *p* < 0.001) as well as in Stage 2 than in Stage 3 (GLMM: *z* = −4.731, *p* < 0.001). The group assignment of the dogs did not influence their success in Stages 2 and 3, but in Stage 1 they performed better when they had to choose the owner's face in contrast to being rewarded for choosing the other person (GLMM: Stage 1: *z* = 2.398, *p* = 0.016; Stage 2: *z* = −0.65, *p* = 0.52; Stage 3: *z* = 0.87, *p* = 0.38). Moreover, dogs showed an increasing number of correct choices across the first three sessions in Stage 2 but not in Stages 1 and 3 (GLMM: Stage 1: *z* = 1.07, *p* = 0.29; Stage 2: *z* = 2.112, *p* = 0.035; Stage 3: *z* = 0.14, *p* = 0.89).

### Transfer from one stage to the next

In Stage 1, the dogs as a group chose the S+ face above chance in the first ten trials (one-sample *t*-test: Stage 1: *t*_14_ = 2.391, *p* = 0.031). In Stages 2 and 3, however, the dogs performed at chance level in their first ten trials (one-sample *t*-test: Stage 2: *t*_11_ = −0.52, *p* = 0.61; Stage 3: *t*_9_ = 1.40, *p* = 0.19). Actually, they dropped from an average of 88% correct choices in their last session of Stage 1 to an average of 48% in their first session of Stage 2. The successful dogs of Stage 2 dropped from an average of 81% correct choices in their last session of this stage to an average of 56% in their first session of Stage 3. There was no difference between Stages 2 and 3 in the number of correct choices the dogs made in their first 10 trials (GLMM: *z* = −1.34, *p* = 0.18).

### Side bias

Over all animals and sessions, we did not find a side bias in any of the three stages (one-sample *t*-test: Stage 1: *t*_14_ = 0.19, *p* = 0.85; Stage 2: *t*_12_ = −1.32, *p* = 0.21; Stage 3: *t*_9_ = 0.16, *p* = 0.88). However, at the individual level over all sessions and stages, seven dogs had a preference for the left side (binomial: Baris: *p* = 0.025; Caya: *p* < 0.001; Flag: *p* < 0.001; Ivi: *p* < 0.001; Jamil: *p* < 0.001; Loki: *p* = 0.001; Marty: *p* = 0.003) and five dogs for the right side (binomial: Aico: *p* < 0.001; Cap: *p* = 0.002; Flamme: *p* < 0.001; Lucy: *p* < 0.001; Nessie: *p* < 0.001). Importantly, while the successful dogs did overcome the side bias, those dogs that failed in Stage 2 or 3 seemed to get stuck in this kind of last resort strategy. For instance, in Stage 2 the dog Flamme always went to the right side for seven sessions in a row but then successfully overcame this habit and eventually learned the discrimination. In Stage 3, however, she always went to the right side for 23 sessions in a row, and the training was then terminated.

## Discussion

In a nutshell, we found that (a) with one exception all dogs were able to discriminate between the owner and another familiar person when they made their heads visible through holes in the box; (b) two thirds of the dogs (10 of 14) mastered the task after a while when, instead of real heads, life-sized pictures of the heads were presented, and (c) only a small minority of dogs (2 of 10) was successful when instead of full heads only (life-sized) pictures of the internal parts of the faces (‘balaclava mode’) were presented.

These results indicate that (a) dogs are able to discriminate familiar humans on the basis of *visual* information from the heads or the faces only; (b) discrimination is difficult when only the inner parts of the faces (eyes, nose and mouth) are visible; (c) they can discriminate not only by differential (preferential) looking but also by making active choices, i.e. by approaching and touching S+ with their noses.

How can we explain that all but two dogs failed to (a) generalize across all three stages and (b) were unable to switch discriminative strategies and re-learn the task? There are several possible answers to that, in terms of perception, feature learning, methodological problems and confusion.

The present experiments may be challenging for dogs with respect to *perception* in at least two different ways, problems of visual acuity and problems with static, 2-D pictures. Especially for face recognition, it is necessary to decipher the tiny details of human faces reflecting the identity of the human person. Although domestic dogs have a larger visual field and higher sensitivity to motion signals than humans, their visual acuity – the ability to see the details of an object separately and unblurred – is up to four times lower than in humans (Miller and Murphy, 1995, Murphy et al., 1997). So if, for instance, a person with normal vision could distinguish the details of a face from 23 m away, normal dogs could do that from only 6 m away. But as the distance in our study was only 1.5 m, finding perceptual differences between the faces seems not to be a serious problem.

Visual acuity depends on the optical properties of the eye, the retina's ability to detect and process images, and the ability of higher visual pathways to interpret images sent to them. In comparison to their ancestors, wolves, the visual acuity of dogs is worse; their maximum density of ganglion cells is comparably lower (Peichl, 1992). Furthermore, among dogs there are significant differences in the distribution of retinal ganglion cells between brachycephalic (“short-nosed”) and dolichocephalic (“long-nosed”) dog breeds. It has been hypothesized that brachycephalic breeds have an advantage in terms of visual acuity because ganglion cells occur more centrally in their retina (McGreevy, Grassi, & Harman, 2004). However, the most successful dogs in our study were mesocephalic dogs (Border Collies).

Notably, the pictures of the heads/faces of the humans were presented in real life-size, as in the study by Nagasawa et al. (2011), rather than as miniaturized images on computer screens, as in our study on the categorization of dogs and landscapes (Range et al., 2008). We do not know which features the dogs used as discriminative cues in the present study, but it is possible that those dogs that failed in Stage 3 (only inner parts of the face presented) had used *global* properties of human heads before, like the color or overall brightness or the hairstyle. In a recent study by Valentini (2012), dogs showed a preference for global over local cues (global precedence) in the visual processing of geometrical stimuli. Importantly, these authors presented the pictures in a way quite similar to the way we did (A4 sized images viewed from about two meters).

The use of global features does not, however, explain the difficulty that all dogs encountered in the transition from Stage 1 to Stage 2. Here the main difficulty is likely to be due to the change from real-life to pictorial presentation. It is well known that non-human animals have difficulty with static, two-dimensional (2-D) pictures as representations of real-life objects (Fagot, 2000). Pictures are always abstractions of their 3-D referents and must therefore appear quite different from real objects to most animals (Bovet & Vauclair, 2000). However, such problems can be solved; even pigeons showed evidence of picture–object recognition (Aust & Huber, 2006). Transfer from Stage 1 to Stage 2 does not need the formation of equivalence relations. It could be mediated by simple, invariant 2-D characteristics without recognition of the real 3-D object. For instance, if dogs used the relative difference in brightness or color between the two heads or parts of it (like hair) as a discriminative cue in Stage 1, this difference would have been preserved in Stage 2. The dogs in this study might not have used such invariant 2-D features of the humans immediately, as they showed a dramatic drop in accuracy when pictures of their owner and the other familiar person were suddenly presented. A similar drop to chance performance was shown in the transition from Stage 2 to Stage 3, which involved the change from pictures of the heads (frontal view) to the same pictures with balaclavas (hiding the hair and the chin). We therefore do not know whether the change in presentation mode (from real heads to pictures of them) or a reduction in the amount of discriminative cues was the main problem in the transition from Stage 1 to Stage 2.

It is, of course, possible that the dogs encountered (also) *non-perceptual* problems with the transition from real-life to pictorial presentation. They may have been *puzzled* by the (changed) requirement to touch a paper where before they touched a familiar real person. Indeed, several dogs refused to make a choice and needed special encouragement at the beginning of Stage 2. Accordingly, they did not seem to spontaneously use the image as a reference to the human person, with their behavior (approach and touch) being an arbitrary (instrumental) response. On one hand, this finding is in sharp contrast to a study with baboons and gorillas that mistook the pictorial stimulus for its referent; in a forced two-choice task between banana pictures and real pebble, they chose and ate the banana pictures, suggesting picture–object confusion (Parron, Call, & Fagot, 2008). On the other hand, our results are in line with the finding that dogs have problems using pictures as representational devices; only one of five dogs could fetch the correct object after being presented with a photo of the target object (Kaminski, Tempelmann, Call, & Tomasello, 2009). But here we have to bear in mind that even young children do not initially understand that drawings can depict real objects. They can learn this, however, if adults provide them with the right experiences (Callaghan & Rankin, 2002).

Most dogs recovered in Stage 2, with two of them showing 70% or more correct trials by the second session, and two more in the third session. Only four dogs were discontinued, two (Leah and Jock) rejected further training due to motivation problems and two (Marty and Aico) were unable to relearn the task even within 20 further sessions. So the overall performance of our dog sample in Stage 2 suggests that the learning of the discrimination between pictures of the heads of two familiar humans is within the reach of most dogs.

In contrast, only a fifth of the sample reached the learning criterion in Stage 3 (being at or above 70% correct responses on three sessions). So why did most of the dogs that could learn to discriminate the pictures of the heads (only frontal view) of two humans fail when presented with the same pictures without hair and chin? Do these external features really convey such important information for dogs? Interestingly, sheep also had problems discriminating human faces on the basis of the internal (face) features. In comparison to the same faces with only the external features visible, their discrimination accuracy was much worse (Peirce et al., 2001). The authors argued that the internal features of human faces were of very little help to them and that their configuration was certainly not important. Also, the fact that the sheep could better discriminate between human faces of different gender than between male faces points to the differences in hairstyles as discriminative cues (Peirce et al., 2001).

In our study with dogs, all owners and non-owners who served as stimuli were women. Still, the hairstyles of women may be salient enough to be used as discriminative cues (see Fig. 2). It is therefore very likely that all but two dogs used external features like hairstyle to solve the discrimination task of Stage 2 (and perhaps also Stage 1) and failed to switch to the use of internal features in Stage 3. Their failure to solve the discrimination task became evident when they switched to a last resort strategy, by going repeatedly to the same side. This side bias, however, is only a symptom and not the cause of their failure to find sufficiently discriminative cues.

To summarize, the pattern of performance of our modestly large dog sample suggests that dogs *in principle* are able to discriminate between two human faces from a distance under even the most difficult condition (static, 2D-representations of internal features only). Nevertheless, most dogs seem to prefer using simpler discrimination strategies, by focusing on salient, global features of their human partners. In the everyday situation, the *identity* of a human person can be assessed by using a huge bundle of features, not only from the face and not only in the visual domain. Olfactory and auditory cues may be much more distinctive or easier to assess for dogs, and if vision is involved, movement is also a strong candidate. The face of their human partners may serve different functions, most probably in the socio-communicative domain.
